# Supplementary material for: Diastereoselective Synthesis of (–)-6,7-Dimethoxy-1,2,3,4-tetrahydroisoquinoline-1-carboxylic Acid via Morpholinone Derivatives
Source: Molecules. 2023 Apr 4;28(7):3200. doi: 10.3390/molecules28073200 (PMC10095930; doi:10.3390/molecules28073200)

Supporting Information

for

Diastereoselective Synthesis of (–)-6,7-Dimethoxy-1,2,3,4-tetrahydroisoquinoline-1-carboxylic Acid via Morpholinone Derivatives

Maria Chrzanowska*, Agnieszka Grajewska* and Maria D. Rozwadowska

Table of Contents

^{1}H NMR and ^{13}C NMR of (*R*)-(–)-*N*-(2,2-diethoxyethyl)-2-phenylglycinol **(47)**……………………………..……………………………3-4

^{1}H NMR, ^{13}C NMR and ^{13}C DEPT NMR of (3*R*, 5*R*)-(–)-4-(2,2-diethoxyethyl)-3-(3,4-dimethoxyphenyl)-5-phenyl-1,4-oxazin-2-one **(48)**...…5-7

^{1}H NMR, ^{13}C NMR and ^{13}C DEPT NMR of (3*S*, 5*R*)-(+)-4-(2,2-diethoxyethyl)-3-(3,4-dimethoxyphenyl)-5-phenyl-1,4-oxazin-2-one **(49)**…8-10

^{1}H NMR and ^{13}C NMR of (*R*)-(–)-*N*-(2,2-diethoxyethyl)-3,4-dimethoxyphenylglycine **(50)**…………………………………………….…...….11-12

^{1}H NMR and ^{13}C NMR of (*R*)-(–)-6,7-Dimethoxy-1,2,3,4-tetrahydroisoquinoline-1-carboxylic acid **(1)**………………………..…………13-14

^{1}H NMR of (*R*)-(–)-*N*-(2,2-diethoxyethyl)-2-phenylglycinol **(47)**

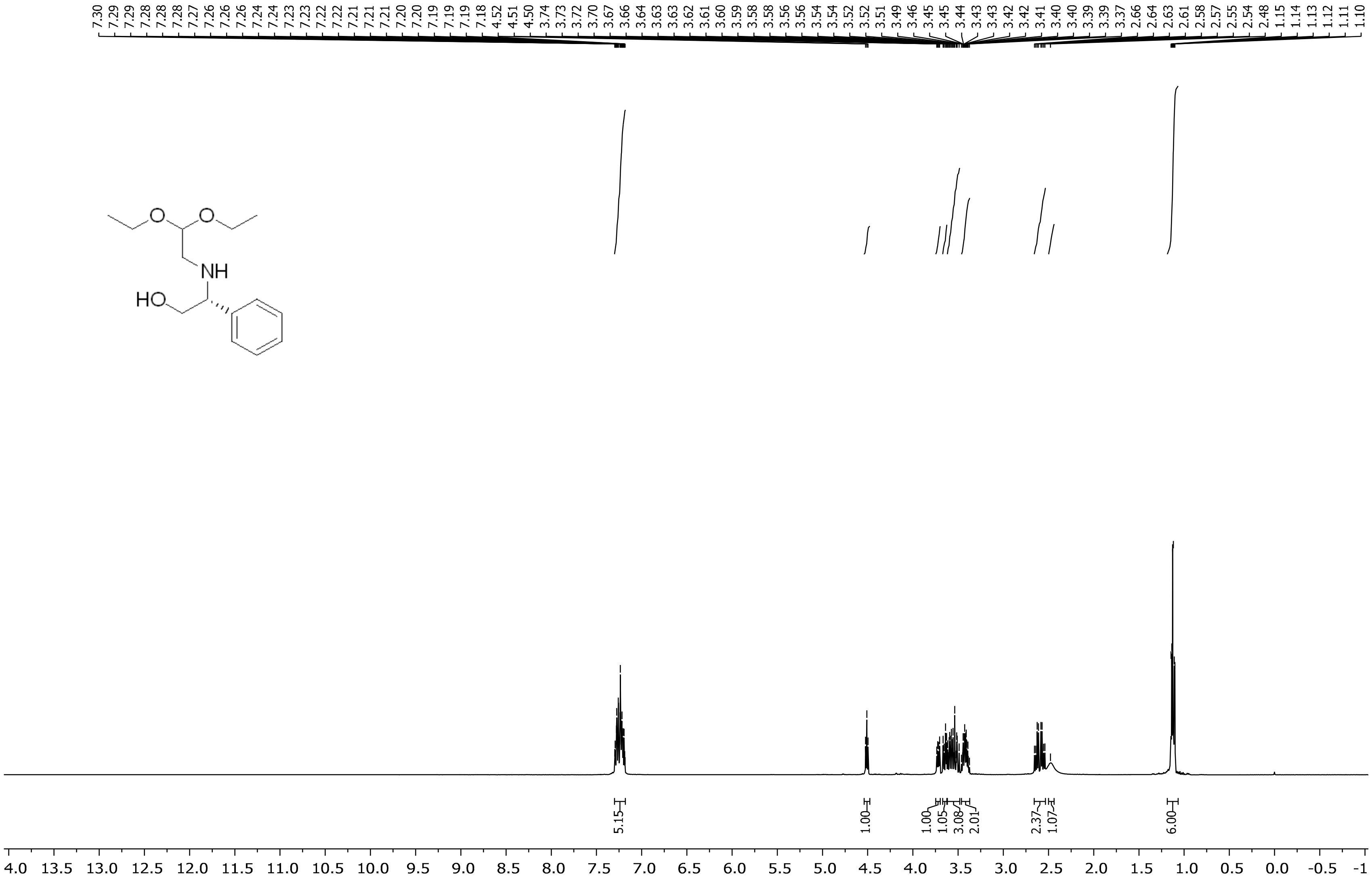

^{13}C NMR of (*R*)-(–)-*N*-(2,2-diethoxyethyl)-2-phenylglycinol **(47)**

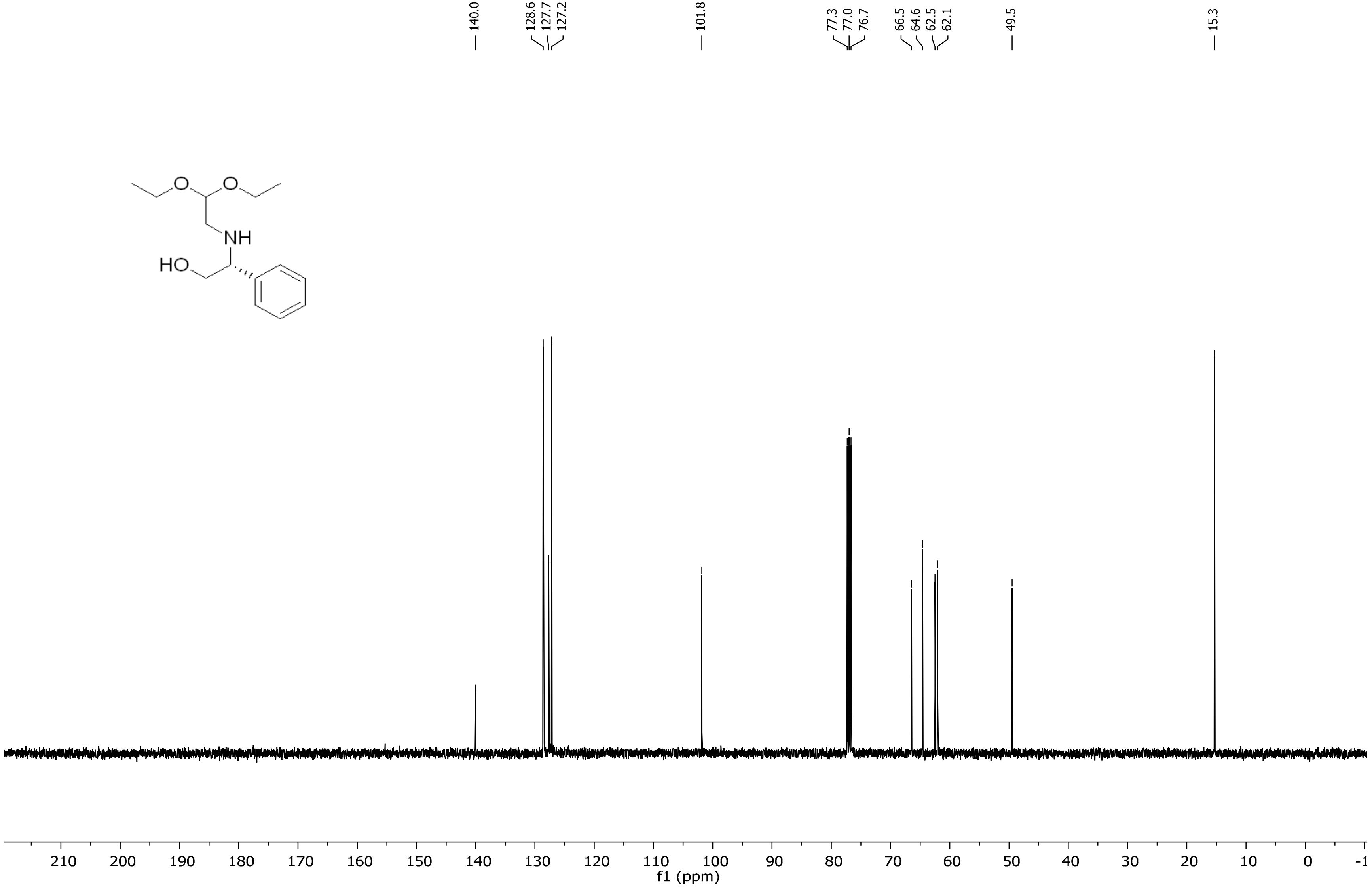

^{1}H NMR of (3*R*, 5*R*)-(–)-4-(2,2-diethoxyethyl)-3-(3,4-dimethoxyphenyl)-5-phenyl-1,4-oxazin-2-one **(48)**

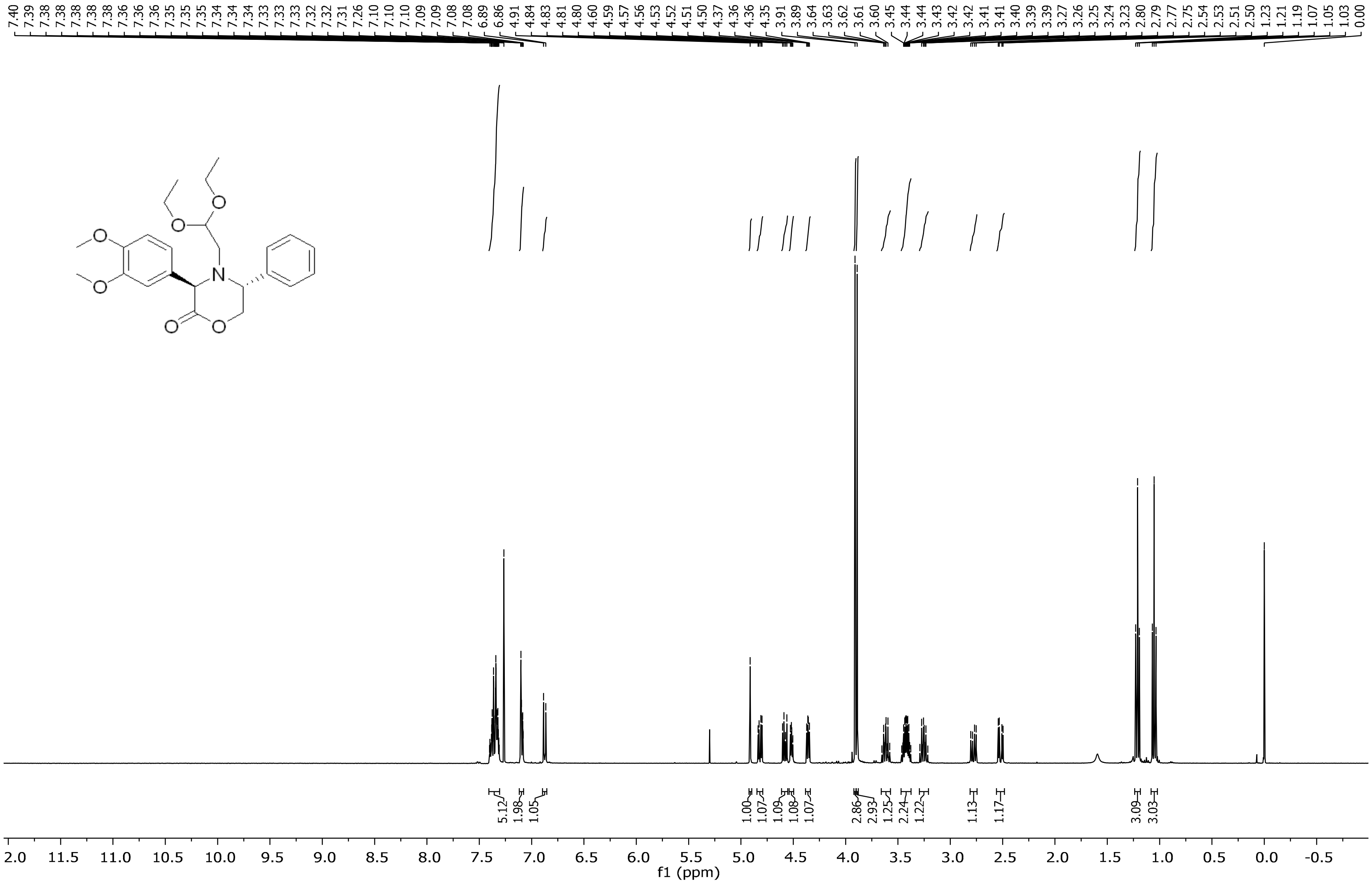

^{13}C NMR of (3*R*, 5*R*)-(–)-4-(2,2-diethoxyethyl)-3-(3,4-dimethoxyphenyl)-5-phenyl-1,4-oxazin-2-one **(48)**

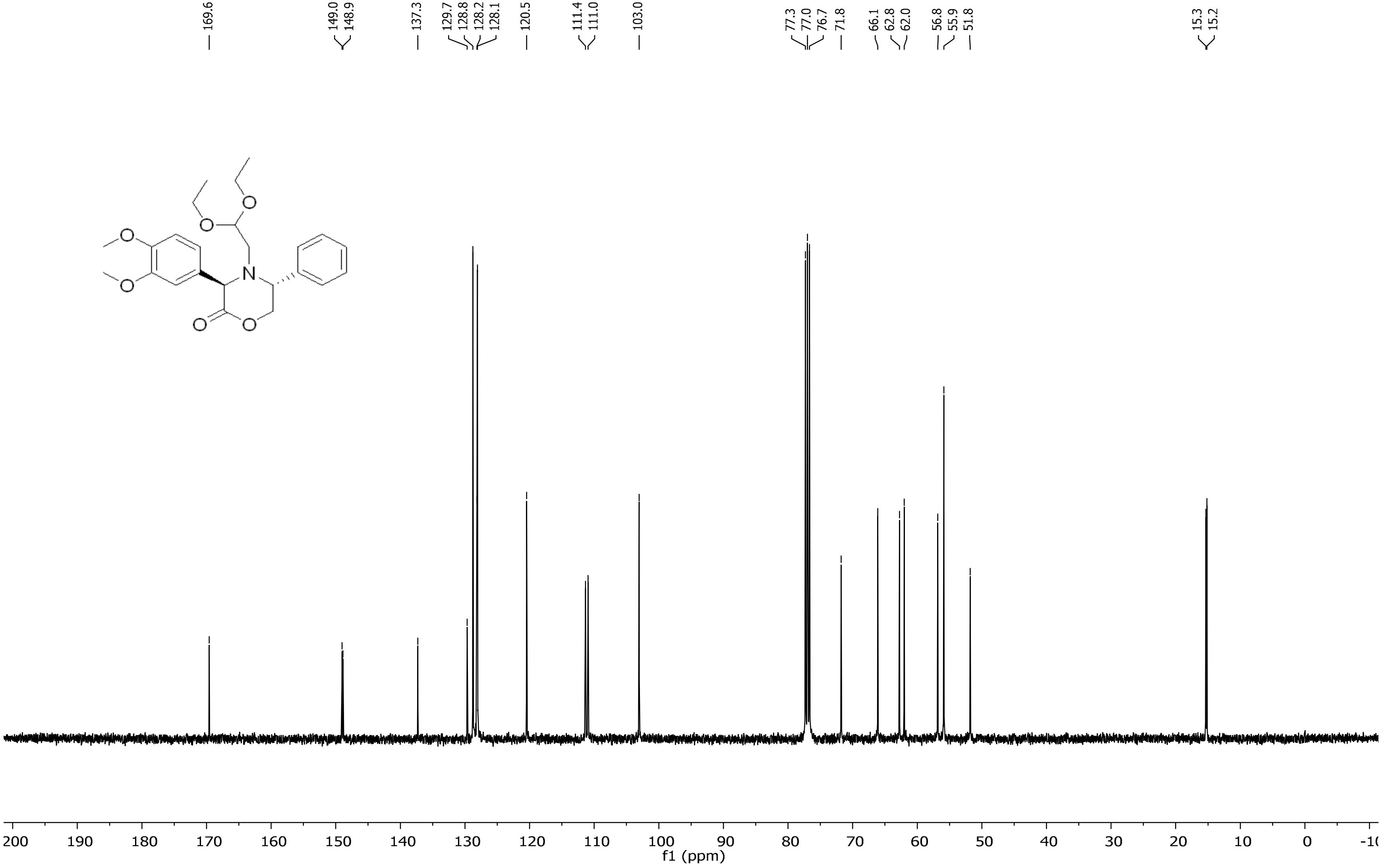

^{13}C DEPT NMR of (3*R*, 5*R*)-(–)-4-(2,2-diethoxyethyl)-3-(3,4-dimethoxyphenyl)-5-phenyl-1,4-oxazin-2-one **(48)**

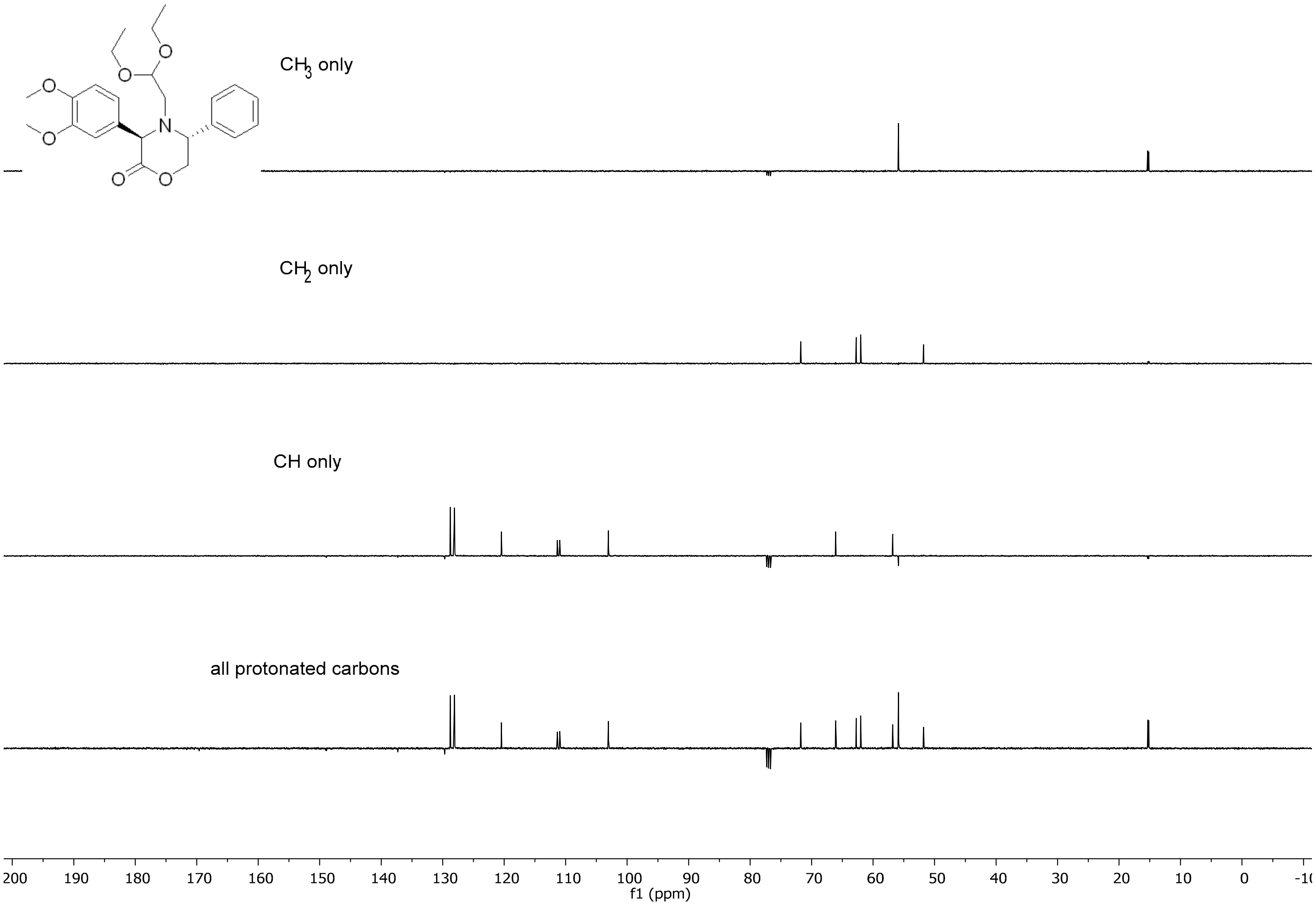

^{1}H NMR of (3*S*, 5*R*)-(+)-4-(2,2-diethoxyethyl)-3-(3,4-dimethoxyphenyl)-5-phenyl-1,4-oxazin-2-one **(49)**

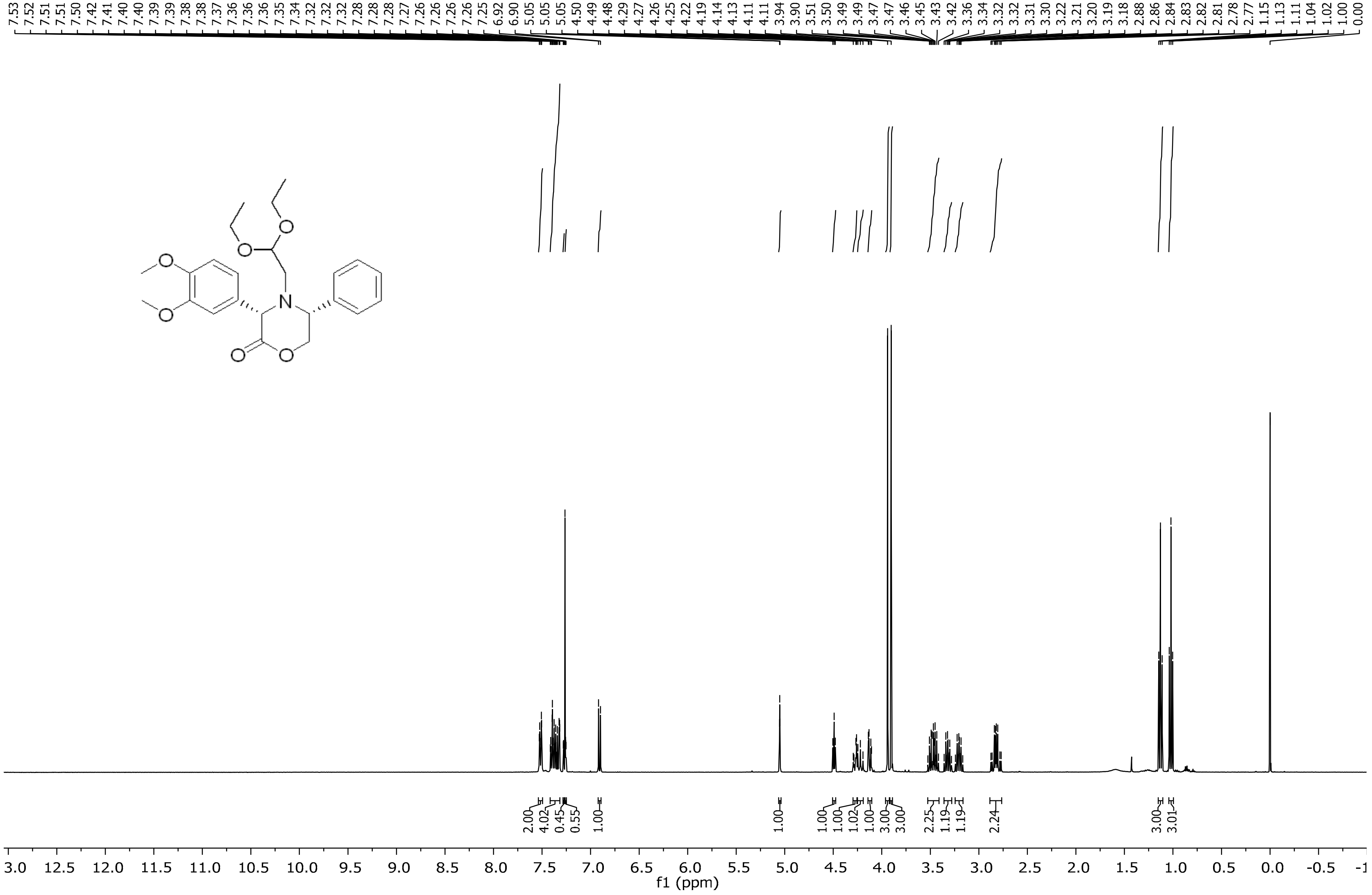

^{13}C NMR of (3*S*, 5*R*)-(+)-4-(2,2-diethoxyethyl)-3-(3,4-dimethoxyphenyl)-5-phenyl-1,4-oxazin-2-one **(49)**

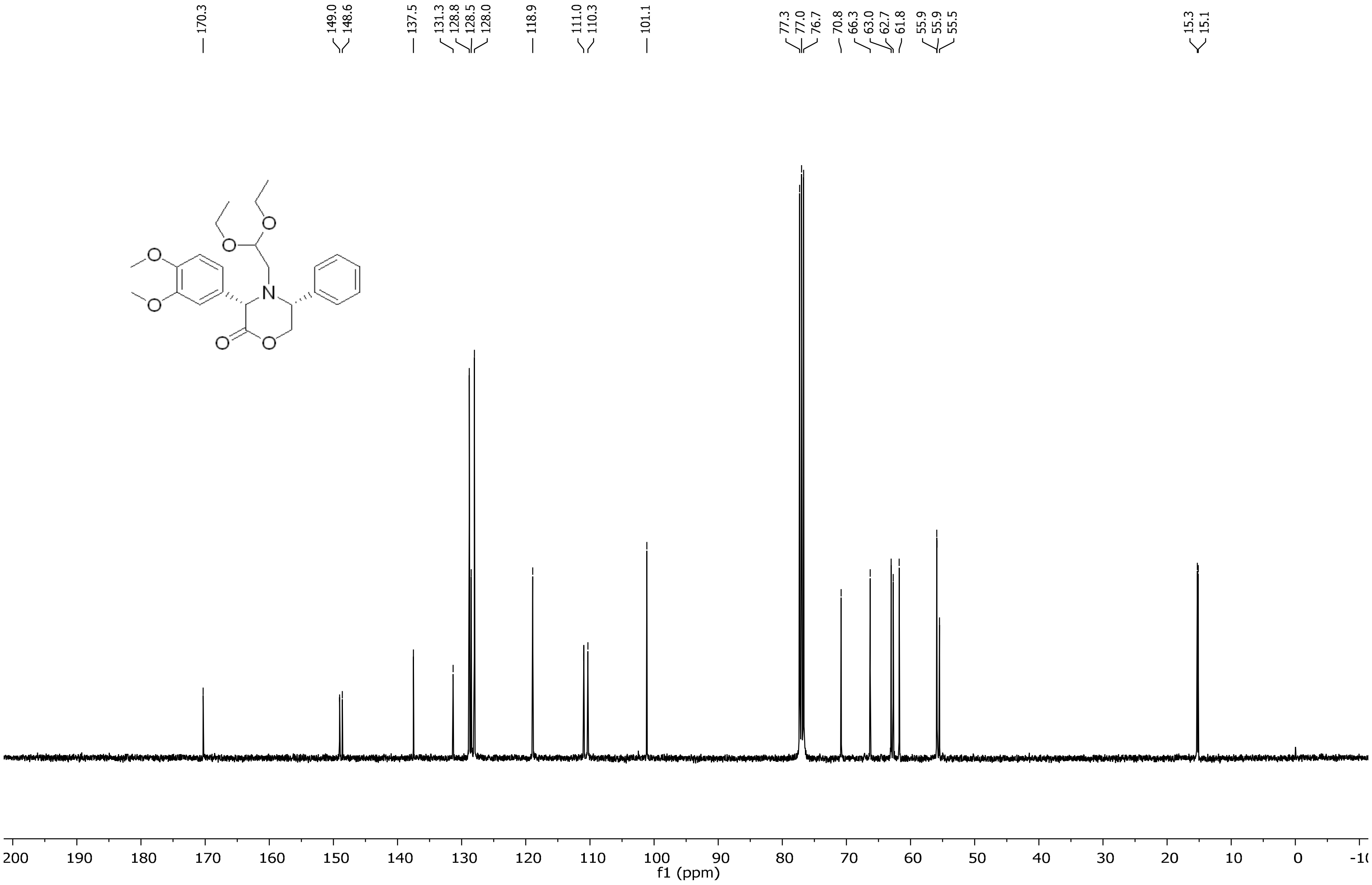

^{13}C DEPT NMR of (3*S*, 5*R*)-(+)-4-(2,2-diethoxyethyl)-3-(3,4-dimethoxyphenyl)-5-phenyl-1,4-oxazin-2-one **(49)**

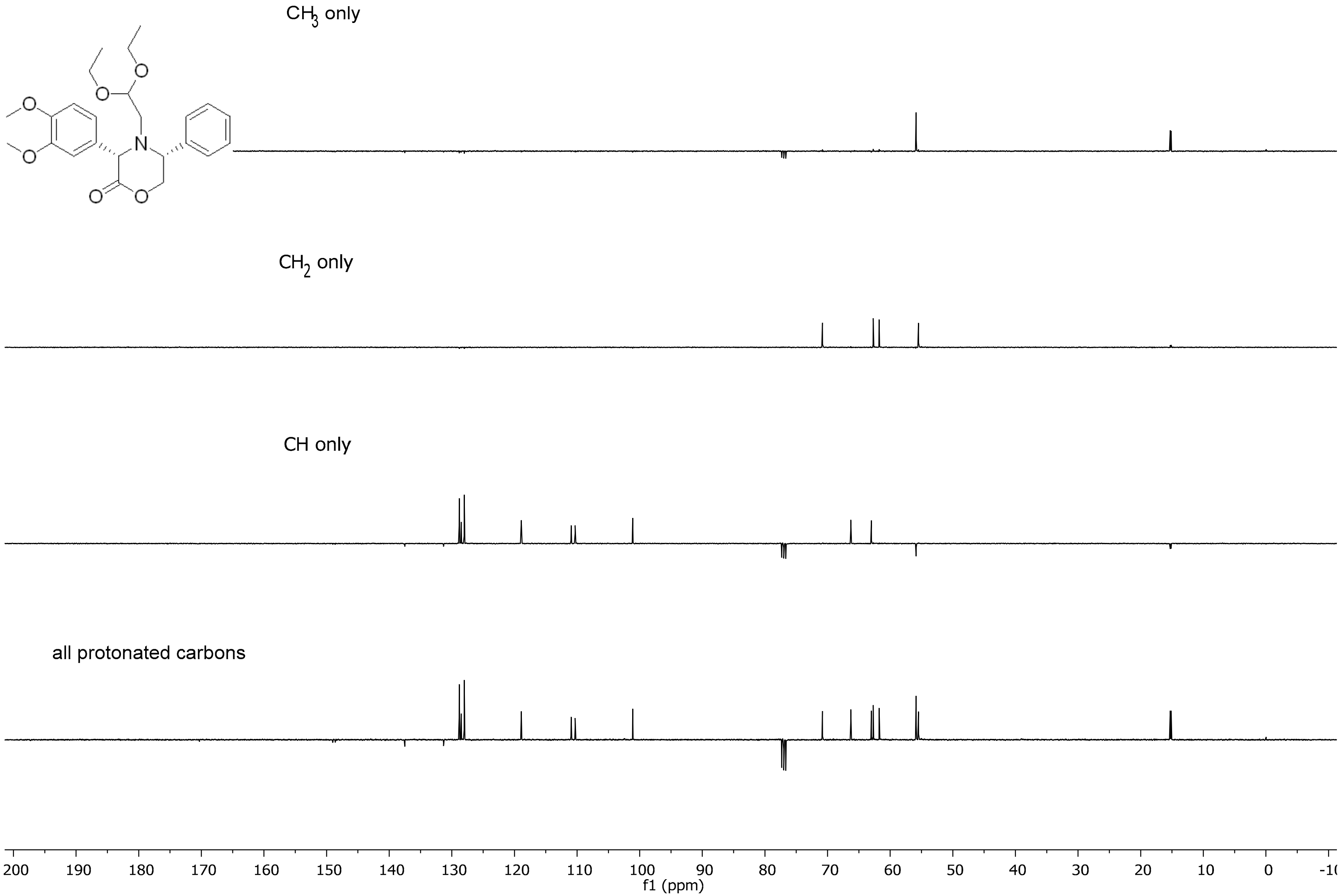

^{1}H NMR of (*R*)-(–)-*N*-(2,2-diethoxyethyl)-3,4-dimethoxyphenylglycine **(50)**

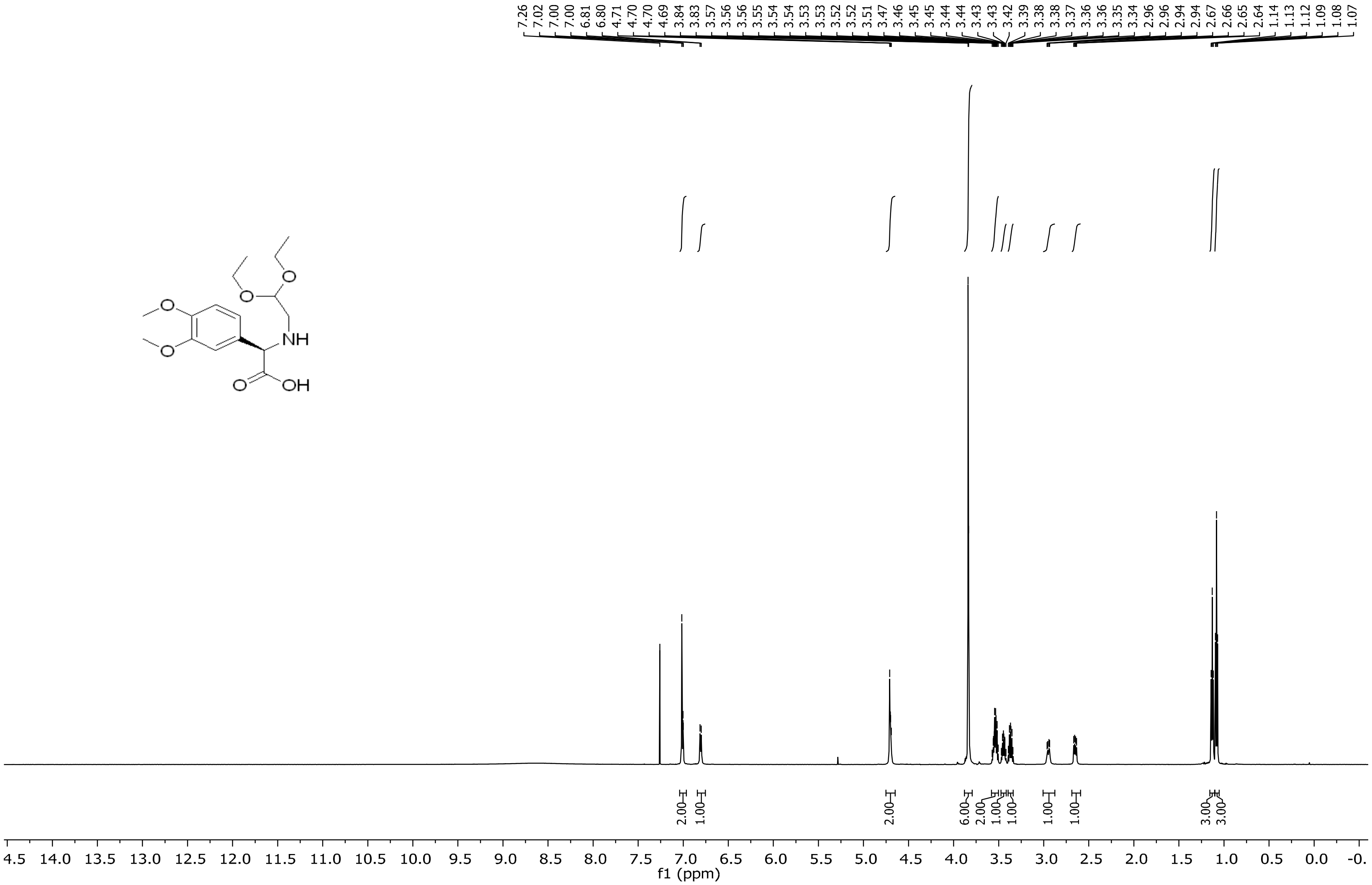

^{13}C NMR of (*R*)-(–)-*N*-(2,2-diethoxyethyl)-3,4-dimethoxyphenylglycine **(50)**

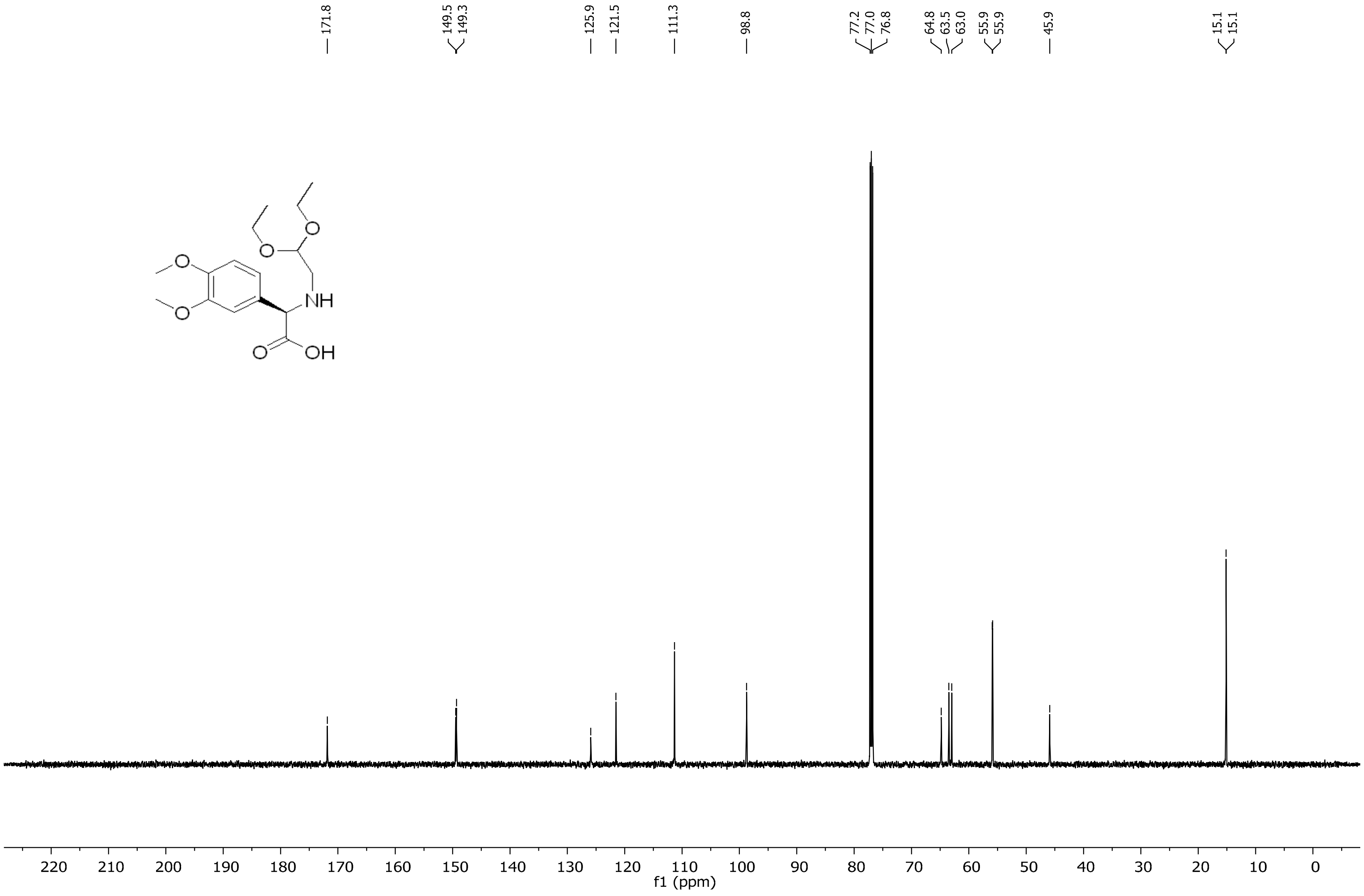

^{1}H NMR of (*R*)-(–)-6,7-Dimethoxy-1,2,3,4-tetrahydroisoquinoline-1-carboxylic acid **(1)**

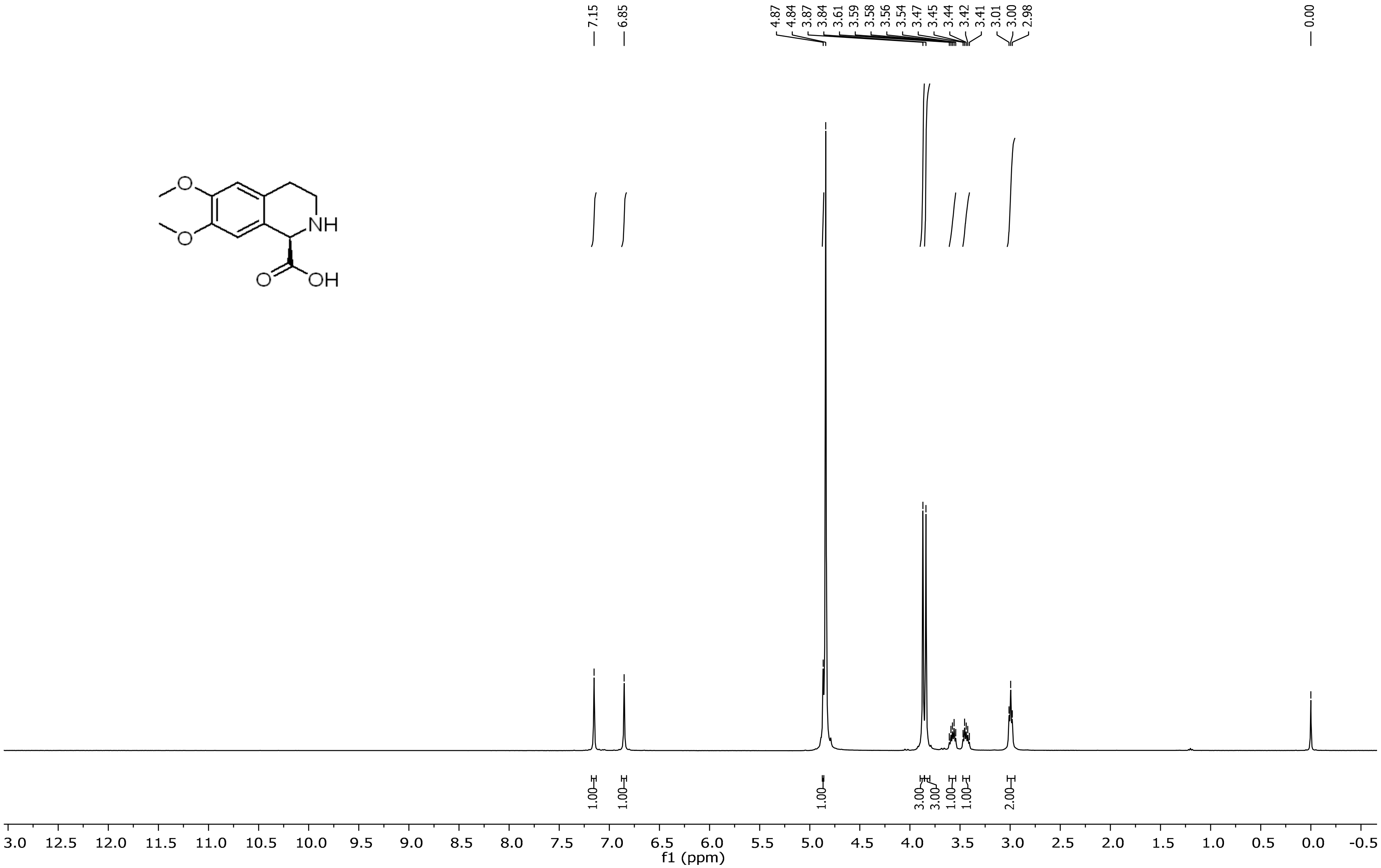

^{13}C NMR of (*R*)-(–)-6,7-Dimethoxy-1,2,3,4-tetrahydroisoquinoline-1-carboxylic acid **(1)**

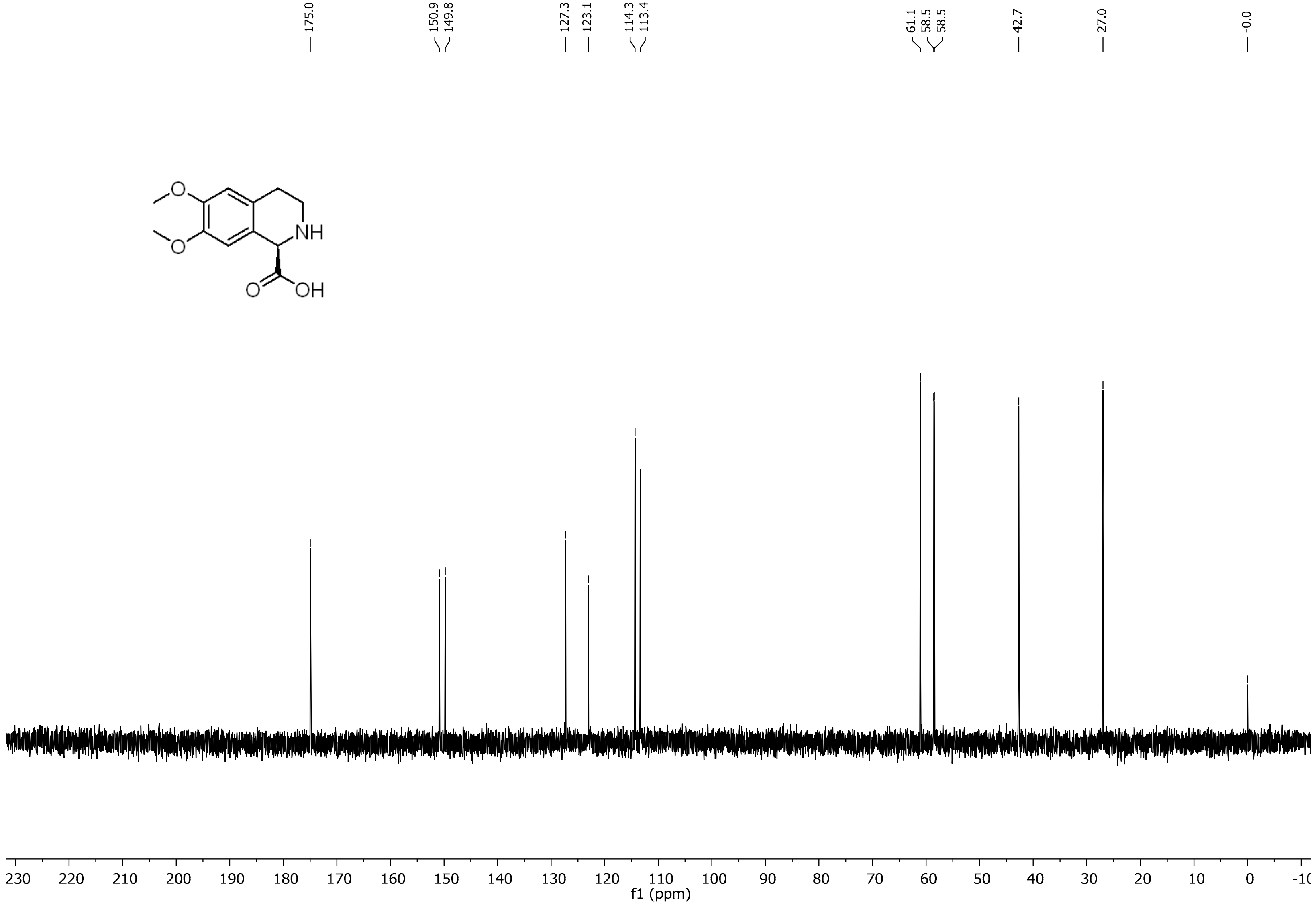

Supplement: Supplementary file 1 [file molecules-28-03200-s001.zip › molecules-2283778-supplementary.pdf]
